# An Intermediate Incubation Period and Primitive Brooding in a Theropod Dinosaur

**DOI:** 10.1038/s41598-018-30085-6

**Published:** 2018-08-20

**Authors:** David J. Varricchio, Martin Kundrát, Jason Hogan

**Affiliations:** 1 Montana State University, Earth Sciences, Bozeman, MT 59717 USA; 2 Pavol Jozef Safarik University, Technology and Innovation Park, Center for Interdisciplinary Biosciences, Kosice, SK-04154 Slovakia

## Abstract

Non-avian dinosaurs such as oviraptorosaurs and troodontids share several important reproductive characters with modern birds, including eggshell microstructure and iterative egg production. Nevertheless, debate exists concerning their incubation strategies. Here we estimate incubation period for the troodontid, *Troodon formosus*, by examining a near-term embryonic tooth. Synchrotron scanning and histologic thin sections allowed counting of daily (von Ebner) growth lines. The tooth preserves 31 intact lines with an average spacing of 3.3 ± 0.96 μm. Adding 8 more for the missing crown tip gives a total age of 39 days. Modern crocodilians begin to establish their functional dentition at approximately 47% through incubation. Thus, this tooth age suggests a *Troodon* incubation period of 74 days, falling midway between avian (44.4 days) and reptilian (107.3 days) values predicted by the *Troodon* egg mass (314 g). An accelerated incubation relative to modern reptiles supports brooding and concurs with a suite of features in oviraptorosaurs and troodontids (sequential laying, large complex clutches, and precocial young) that appear dependent upon both adult body and incubation temperatures elevated over ambient conditions. However, the largely buried condition of *Troodon* clutches may have prohibited efficient brooding, necessitating longer incubation than that of modern birds with fully exposed eggs.

## Introduction

**Figure 1:**
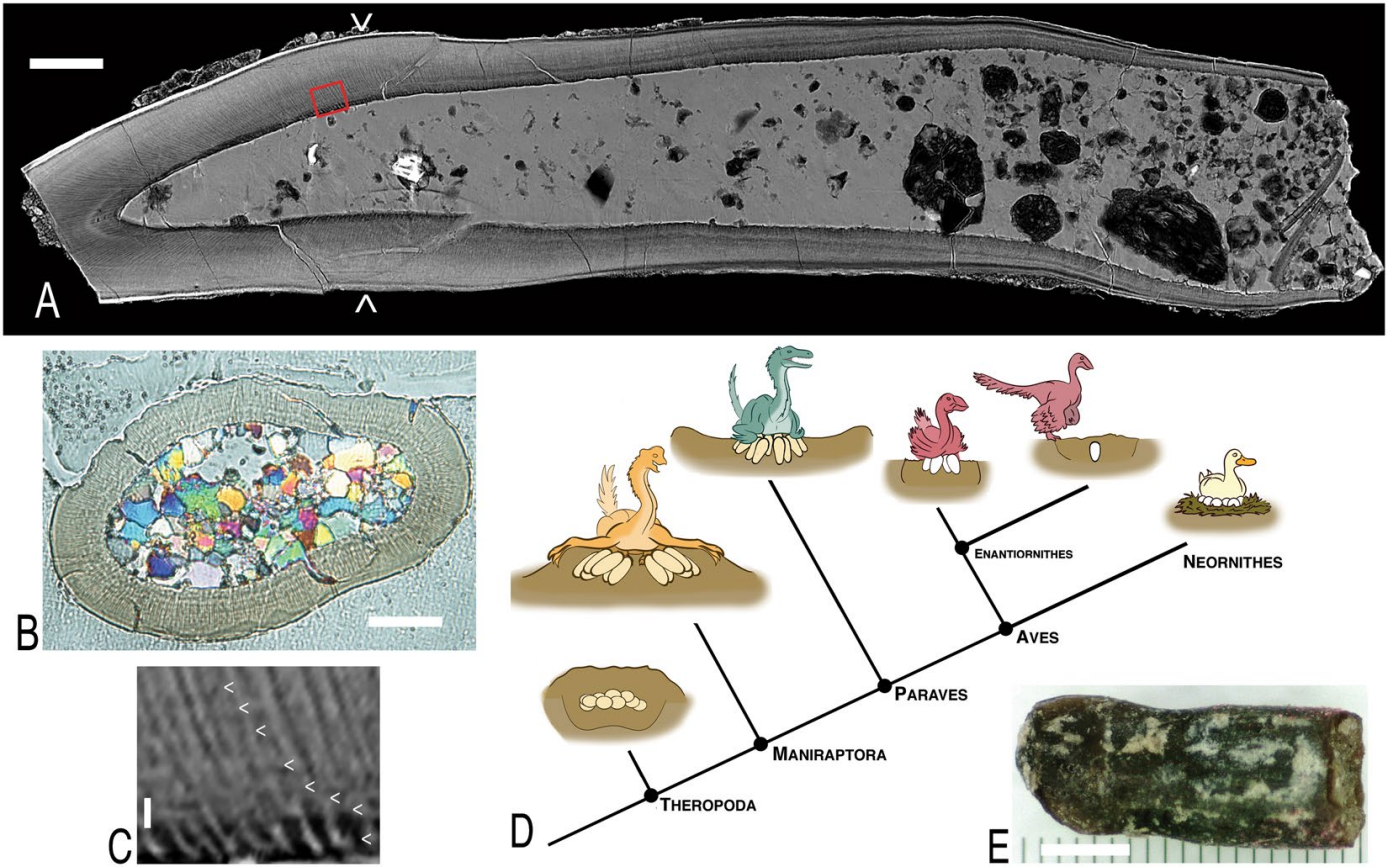
(**A**–**C**,**E**) Embryonic *Troodon* tooth of MOR 246-11 in lingual view (**E**), longitudinal section produced from synchrotron scanning (**A**), and cross sectioning from histologic thin sectioning (**B**). Carets and box in A mark, respectively, the approximate position of the cross section in B and enlarged section in C showing a close-up of daily growth lines. Scale bars = 100 µm in A and B, 5 µm in C, and 0.5 mm in E. (**D**) phylogeny showing hypothesized incubation methods among theropod dinosaurs with burial of clutches as the primitive condition among theropods and likely most dinosaurs; brooding of more derived and partially exposed clutches among maniraptoran dinosaurs such as oviraptorosaurs and troodontids and perhaps some enantiornithine birds of the Mesozoic, and finally eggs brooded completely free of sediment burial in Neornithes. Modified from Varricchio and Jackson^34^ with artwork by Danny Anduza.

The modern bird egg clearly traces its ancestry into non-avian theropod dinosaurs such as oviraptorosaurs and troodontids (Fig. 1D). These maniraptoran dinosaurs share with modern birds: eggs with hard, calcitic shells with narrow shell units, a second structural layer of vertical prisms, sparse and narrow pores, late calcium absorption (“cratering”) of the mammillae by the developing embryo, and at least some textural development within the continuous layer that paleontologists refer to as “squamatic structure”^1–8^. In comparison to oviraptorosaurs^9^, troodontids like *Troodon*, share additional features found in most living birds including a third, external shell layer^10^, an absence of eggshell ornamentation, and a more asymmetrically shaped egg^1,11^. Further, the common eggshell microstructure and within-clutch egg pairing^12^ in these dinosaurs, as well as an oviraptorosaur adult with two internal eggs^13^, indicate that overall ovary and oviduct function in these dinosaurs matched those of modern birds in producing eggs iteratively at daily or greater intervals, but from two active reproductive tracts^12–14^.

Nevertheless, much debate exists concerning incubation strategies in these non-avian dinosaurs and whether it is homologous to that of modern birds. Parental care of eggs and brooding with active transfer of heat to eggs have been inferred for both oviraptorosaurs and troodontids. For oviraptorosaurs, the most convincing evidence are clutch-associated adults preserved, in some cases, in life-like postures over the eggs^15–17^. Although two clutch-associated adults are known in troodontids^12,18^, more compelling evidence includes an intact *Troodon* nesting trace with clutch and low overall egg porosity^19,20^. Among modern egg-layers, egg porosity corresponds closely to nesting environments, with low values associated with exposed conditions and brooding in the case of birds^21,22^. Counterarguments to these interpretations favor incubation from soil burial^23,24^ and reptile-like nest attendance or guarding based on the limited contact between adult and eggs^21,23–25^, the presumed inefficiency of transferring body heat to a partially buried clutch, the absence of egg rotation^26,27^, intermediate and perhaps ambiguous porosity of oviraptorosaur eggs^21,22^, uncertainty about adult body temperature, and the size disparity between the fairly compact *Troodon* clutch and the larger, surrounding nest structure^25^.

Two recent studies^28,29^ experimentally tested and outlined a method to determine the age of dinosaur embryos based on growth-line counts in their teeth. Later, Erickson *et al*.^30^ used growth-line counts in embryonic teeth to determine incubation periods for dinosaurs. Based on daily incremental lines (von Ebner lines), they aged embryonic teeth in two dinosaur taxa and then calculated their incubation periods based on estimates of the establishment of hatchling functional dentitions. Both species examined, *Hypacrosaurus* and *Protoceratops*, exhibited long, reptile-grade incubation periods^30^ with the estimated values 209% and 208%, respectively, of the incubation period predicted for an avian egg of similar size using the regression equation of Deeming *et al*.^31^. These values far exceed those estimates generated for dinosaurs when modeled using a metabolic mass gain parameter, a measure of growth, based on avian embryos^32^ and potentially supported by avian-like tissues within embryonic bone^33^. But, they are consistent with the high eggshell porosity found in the vast majority of dinosaur eggs and the likely associated buried incubation mode^21^. The predicted values are more similar, 111% and 83%, respectively, to incubation periods estimated by a modern reptilian model^31^.

Here we apply these embryonic aging methods^28–30^ to test the incubation strategy of the troodontid, *Troodon formosus*, a dinosaur, like oviraptorosaurs, hypothesized to have a reproductive mode (Fig. 1D) intermediate between reptiles with fully buried clutches produced en masse and modern birds with iteratively produced eggs incubated free of sediment^12,34^. Regressions of egg mass versus incubation period from modern vertebrates provide estimates of expected values for these two incubation endpoints. Using a *Troodon* egg mass of 314 g^20^ and equations of Deeming *et al*.^31^, *Troodon* would be expected to have an incubation period of 107.3 days with reptilian soil burial but only 44.4 days for avian sediment-free brooding.

## Methods

We scanned via synchrotron microtomography and examined an embryonic *Troodon* tooth from Museum of the Rockies (MOR) 246-11. MOR 246 represents a clutch of 19 partial eggs, most of which contain some embryonic remains; egg #11, MOR 246-11, preserves a partially articulated embryo and some associated elements^11^. The tooth scanned in this study was a disarticulated tooth from this egg and embryo. This specimen comes from the Campanian Upper Cretaceous Two Medicine Formation of Montana, the same unit yielding eggs, clutches, and nest structure for *Troodon*. The species *Troodon formosus* Leidy 1856^35^ was originally established on a tooth from the Campanian Judith River Formation of Montana. In 1987, Currie revised the taxon and synonymized several subsequently named species into *T*. *formosus*^36^. In 2017 van der Reest and Currie^37^ recognized that *T*. *formosus* as defined by Currie^36^ included two taxa, one of which they named *Latenivenatrix mcmasterae* and the other they referred to *Stenonychosaurus inequalis*. Given that the latter had already been synonymized into the senior *T*. *formosus*^36^ and remained unused for 30 years, *Troodon formosus* remains the proper name for this taxon, exclusive of *L*. *mcmasterae*, and we continue to use it here.

The MOR 246-11 tooth (Fig. 1E), currently missing the very tip of its crown, measures 1.9 mm long, 0.8 mm wide by 0.36 mm. Likely, with a complete crown, the tooth would be nearly 2.1 mm long. This tooth is similarly sized to other fully erupted and *in situ* teeth for this embryo^11^. The ossification of most skeletal elements^11^, as well as the histology of a similarly sized embryo, MOR 246-1^33^, from the same clutch indicate that this embryo (MOR 246-11) was in a late stage of development and close to hatching. Consequently, this tooth likely represents one from the final hatching compliment.

Scanning was performed at the beamline ID19 of European Synchrotron Radiation Facility in Grenoble (France). The scan was collected with propagation phase contrast synchrotron microtomography using a monochromatic beam with an energy approximately 30 keV. The scanned data of the complete specimen has an isotropic voxel size of 0.6 µm. The reconstructed slices were converted into a 16 bit.tif image stack (2046 projections) that was concatenated to obtain a single stack covering the area of interest. To reduce the data size for general anatomical observations, a second version of the reconstructed scan was calculated with 2 × 2 × 2 binning. VG Studio Max version 3.0 (Volume Graphics Inc., Germany) was used for image analysis.

We counted the daily growth lines found in the tooth dentin, lines of von Ebner, using a longitudinal section generated from the synchrotron data (Fig. 1A). To compensate for potentially missing lines in the tooth tip, we first reconstructed the crown based on the preserved trajectories of the enamel, then estimated the number of missing growth lines based on the average spacing of observable lines. For comparative purposes, we measured the spacing of growth lines by hand off enlarged printed images.

To verify observations from the scans, we also thin sectioned the tooth and examined it with light microscopy. For the thin sectioning, two transverse segments were taken from the tooth, both closer to the crown where the pulp cavity accounted for a smaller percentage of the tooth volume than towards the root (Fig. 1B). Although a longitudinal section would have been ideal to compare to the synchrotron scan, the smallest saw blade available on site at just 0.36 mm wide would have taken out too substantial an amount of material as kerf loss. Additionally, the thinnest saw blade tended to bend as test cuts in the epoxy block were made, and even the slightest curve in the cut could have destroyed the tooth sections. Another method considered was to grind down the outside edges of the tooth to end up with a mid point longitudinal section, but it was again decided that the heavy loss of material and single resulting thin section made the procedure too risky. Instead, two transverse sections were taken to ensure that the average distance between von Ebner lines could be verified even if not all lines would be observable in this cross section. The second thinnest saw blade was used to create the transverse sections in order to avoid cut bending. Additional kerf loss resulting from the wider blade was acceptable due to the orientation of this cut. After the initial cut through the specimen we ground each piece towards the apical point of interest, tip inwards for one thin section and root inwards for the other. The thin sections were originally ground to 100 µm and then viewed and imaged every 10 µm until the target thickness of 50 µm. The thin sections were further ground to 35 and 30 µm in an attempt to get beyond the tubule interference, however the visibility of the von Ebner lines was reduced at these thicknesses instead. The clearest visuals of the daily growth patterns came from the 50 µm-thick thin sections. Daily growth lines were observed initially using polarized light microscopy and secondarily with confocal microscopy. The polarized light microscope was a Nikon Optiphot2-POL with a Digital Sight Camera and Prior Optiscan II stage. Nikon BR Software was used for subsequent viewing and analysis. The confocal microscope was an upright Leica SP5 Confocal Laser Scanning Microscope with corresponding Leica objectives.

In order to ensure that the lines visible in the synchrotron generated cross section were not imaging artifacts, corroborating measurements were taken on both standard polarized light and confocal microscopes. The confocal microscope was used in an attempt to see through the noise created by the abundant dentine tubules, though it did not provide significant additional resolution.

Erickson *et al*.^30^ estimated incubation period for dinosaurs assuming that the establishment of their functional dentition conformed to the pattern in modern crocodilians which begins at 42–52% of the total incubation period, values we use here for *Troodon*. Dinosaur values presented by Erickson *et al*.^30^ used the 42% value in order to minimize and be conservative about the long overall incubation periods predicted. Here we present results using the extreme values as well as the average of 47%.

## Results

In contrast to the teeth found in therizinosauroid embryos with crenulated crowns^38^, the *Troodon* crown is smoothly enameled. The longitudinal scan reveals the narrow tooth to consist of a large pulp cavity surrounded by dentine and a very thin exterior of enamel, both of which thicken toward the tooth crown (Fig. 1A). A uniform matrix with some coarser silt-sized clasts appears to fill the pulp cavity. Thin fragments of the very base of the tooth root are broken off and preserved within the bottom portion of the pulp cavity fill. Within the dentine, tubules radiate out ubiquitously and generally appear more visible than the fainter growth lines. Interior growth lines, i.e., those closer to the pulp cavity, were more apparent at roughly the mid height of the tooth, and counting began here. Subsequently, more exteriorly positioned lines could be traced by moving apically. In total, the MOR 246-11 tooth preserves 31 intact growth lines with an average spacing of 3.3 ± 0.96 µm (Fig. 1A,C).

The thin sections of the tooth concur with the synchrotron imaging in revealing a large pulp cavity surrounded by dentine and a very thin exterior of enamel (Fig. 1B). Sparry calcite largely fills the pulp cavity and would appear to represent the uniform matrix of the synchrotron scans. Here too, the closely packed dentine tubules radiate out from the central cavity perpendicular to the daily growth lines and visually predominate the von Ebner lines at every focal depth. High microscope magnifications only exacerbated the issue as their slim focal planes made von Ebner lines more difficult to pinpoint while the tubules remained ubiquitous. Focusing through the tubules was likewise unsuccessful as they were packed densely on top of each other throughout the tooth. We attempted to use a confocal microscope to isolate a more useful field of view, however this yielded practically the same images with only marginally more contrast. Nevertheless, distinct banding of concentric growth lines could be viewed through polarized microscopy. Due to the orientation, the transverse thin sections did not allow every von Ebner line to be visible, however those present maintained the same interline spacing (3.3 μm) as seen in the synchrotron image.

The narrow width of the incremental lines, relative to those in the other dinosaurs^30^, may reflect the high compliment of relatively small teeth (>130) found in the functional dentition of *Troodon*. An estimated eight additional lines would appear to be missing from the crown tip, giving an entire age of 39 days for the tooth. This translates to an incubation period of between 67 and 81 days, or an average of 74 days. In comparison to the periods estimated by Erickson *et al*.^30^ for *Hypacrosaurus* and *Protoceratops*, the average *Troodon* incubation period falls nearly mid way between the predicted avian and reptilian values, 44.4 vs.107.3 days, respectively^31^, the difference being slightly less to the former (a difference of 40%) than the latter (45%). Both minimum and maximum values also fall well within the predicted values. The minimum value (67 days) for *Troodon* incubation would differ by 60% from the reptilian and 34% from the avian periods, whereas the maximum value (81 days) deviates from these times by 32% and 45%, respectively.

## Discussion

The synchrotron imaging and the thin sectioning provide similar quality visual images of the von Ebner lines. However, given the non-destructive nature and greater overall perspective of the tooth provided by the synchrotron scans without any risk of losing the specimen during preparation nor a loss of visual quality of the tooth histology, we would recommend this method over traditional thin sectioning for future work.

The need to account for the missing tip introduces some estimation into the aging of the tooth. However, any error is unlikely to move *Troodon* incubation into either modern avian or reptilian ranges. At the most extreme, if one added no extra days or doubled the number added, estimates clearly not supported by the observed enamel trajectories, predicted incubation periods would be 60 and 90 days, respectively. These values still fall well between those predicted by the modern avian and reptilian models. Potentially, our reconstruction might be off by a few days (e.g. +/−3) giving a range of 68 to 80 days.

The estimated 74 days of incubation for *Troodon* appears to be clearly intermediate between the incubation periods of extant birds and reptiles. For example, the *Troodon* value lies well outside the 95% confidence interval for bird incubation periods, which predicts a maximum value of 61 days for an equivalent-sized egg^39^. Although some birds, e.g. megapodes and procellariforms, exhibit relatively long incubation periods for the size of their eggs, this likely reflects distinctive reproductive attributes. Megapodes rely on vegetation mounds, soil burial, and other non-brooding mechanisms to incubate their eggs^40^. Procellariforms have the longest incubation periods among brooding birds and their incubation model^31^ predicts 68 days for a *Troodon*-sized egg. But they produce a single, large egg and often exhibit egg-neglect, where parents leave the egg unattended for hours to days at a time to feed on scarce marine resources^41–45^. Egg neglect is unlikely to account for the incubation period in *Troodon* as the strategy occurs in birds breeding in remote locations (e.g., islands, cliffs) largely free of terrestrial predators^42–45^. The slow development of superprecocial young in megapodes also appears to contribute to their longer incubation period relative to that of other extant birds^40^. However, hatchling developmental state is unlikely to account for the shortening of incubation as hypothesized here as no clear evidence currently exists to suggest that *Troodon* would differ from other theropod dinosaurs in hatchling condition^46^.

Comparison of *Troodon* incubation with that of modern reptiles is more challenging. First, *Troodon* egg mass far exceeds that of any modern reptile^47,48^ and secondly, the correlation of egg mass and incubation is far weaker for reptiles than for birds. R^2^ values are 0.27 and 0.70, respectively^31^. Among extant crocodilians, several species have incubation periods within the estimated range of *Troodon*, but these possess much smaller eggs, only 22–38% the mass of a *Troodon* egg^47,48^. The few non-brooding, non-archosaurian reptiles with eggs greater than 100 g, all have incubation periods of 90 days or more^47^.

For eggs of similar size, birds typically require shorter incubation periods than most reptiles and this is, in part, a product of incubation temperatures that are 5–8 °C higher^49^. The shortened incubation period in *Troodon* relative to both modern reptiles and the two ornithischian dinosaurs supports the hypothesis that brooding by adult troodontids elevated clutch temperatures sufficiently over environmental conditions and those typical of reptilian incubation. This concurs with suite of features found in both oviraptorosaurs and troodontids (sequential laying, large complex clutches, precocial young, and presumed synchronous hatching) that appear dependent upon both adult body and incubation temperatures elevated over ambient conditions^50^. For example, in most modern birds with large clutches and precocial young, adults refrain from incubating eggs until completion of the clutch^51–53^. Thus, embryos in earlier laid eggs remain at ambient temperatures and in developmental stasis until brooding begins. Brooding begins with the completion of the clutch, raising the embryos to incubation temperatures and synchronizing hatching of the precocial young^51^. Such a hatching mode was likely necessitated by the complexly arranged and stacked egg clutches in troodontids and oviraptorosaurs^50^. Recent stable isotopic work further corroborates this interpretation as it indicates oviraptorosaurs incubated their clutches at temperatures similar to those of extant brooding birds^54^. However, as previously suggested^26,27^, the partial burial of *Troodon* clutches may have represented a less efficient brooding situation, with more heat lost by conductance to the ground and an incubation period substantially longer than that predicted for a brooding avian model with eggs completely sediment free within the nest.

Major evolutionary changes related to reproduction occur within the maniraptoran dinosaur clade (Fig. 1D). In contrast to most other theropods and dinosaurs in general, oviraptorosaurs and troodontids possessed iterative egg production, eggshell microstructure more similar to that of modern birds, elongate eggs much larger relative to adult size, elaborate clutch configurations, brooding^34^, shorter incubation periods and possibly paternal (male only) care^46^. Potentially this shift may reflect selection for fewer, larger young with greater parental investment.

The long incubation time required for dinosaurs like *Troodon* emphasizes the extensive temporal investment made by adults for reproduction. Nest construction, iterative egg-laying of large clutches, and a lengthy brooding period would likely require three months per year or more of nest-site residence. The method to age embryos by their teeth^28–30^ provides a means to quantify incubation period. Although uncertainty exists for when embryonic dinosaurs established their functional dentition and the validity of using the timing in modern crocodilians, the results for two ornithischians^30^ and for *Troodon* in this study appear consistent with nesting habits as inferred from clutches, nesting traces, and eggs^12,19–21^.
